# Supplementary material for: Combining phylogeny and coevolution improves the inference of interaction partners among paralogous proteins
Source: PLoS Comput Biol. 2023 Mar 30;19(3):e1011010. doi: 10.1371/journal.pcbi.1011010 (PMC10089317; doi:10.1371/journal.pcbi.1011010)
Supplement: S1 Text — (PDF) [file pcbi.1011010.s001.pdf]

Supporting Information
Combining phylogeny and coevolution improves the inference of interaction partners among paralogous proteins

Carlos A. Gandarilla-Pérez[1,2], Sergio Pinilla[2,3,¤], Anne-Florence Bitbol[4,5*], Martin Weigt[2*]

1 Facultad de Física, Universidad de la Habana, San Lázaro y L, Vedado, Habana, Cuba
2 Sorbonne Université, CNRS, Institut de Biologie Paris-Seine, Laboratoire de Biologie Computationnelle et Quantitative (LCQB, UMR 7238), Paris, France
3 Sorbonne Université, CNRS, Institut de Biologie Paris-Seine, Laboratoire Jean Perrin (UMR 8237), Paris, France
4 Institute of Bioengineering, School of Life Sciences, École Polytechnique Fédérale de Lausanne (EPFL), Lausanne, Switzerland
5 SIB Swiss Institute of Bioinformatics, Lausanne, Switzerland

¤Current address: Heuritech, Paris, France
* anne-florence.bitbol@epfl.ch (AFB); martin.weigt@sorbonne-universite.fr (MW)

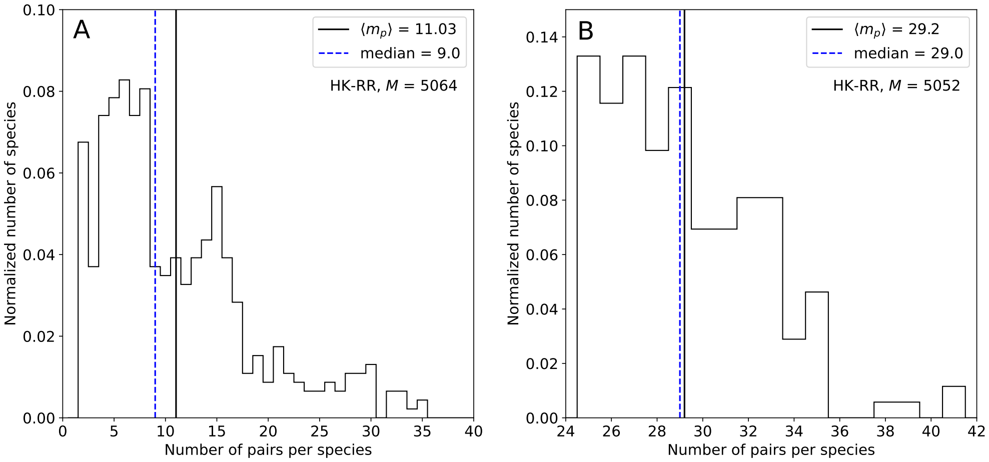

Fig A. Histograms of paralog multiplicities for the two HK-RR datasets used in the paper (standard dataset used e.g. in Fig 4, and dataset with more paralogs per species used in Fig 5A, cf. *Materials and methods*), which have mean paralog number 11.03 (A) and 29.2 (B). We see that the paralog numbers vary widely from two (as imposed by the dataset construction) and 41.

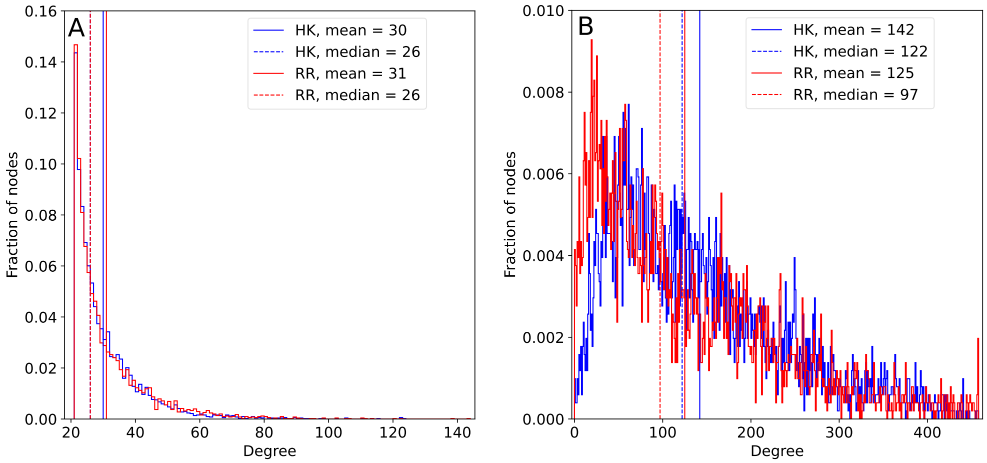

Fig B. Degree distribution of sequence-similarity networks. (A) For kNN networks (here with $k = 21$, blue for HK, red for RR), the minimum number of links is k. We find that half of the nodes have degrees smaller or equal than about 30, and the other half may have up to degree 140. (B) The degree distributions for the orthology networks are more skewed and much broader than for kNN networks, resulting in larger differences between median and mean degrees. There is a peak corresponding to the maximum possible number of links in this kind of graph, which is the number of total species minus one. The standard dataset used e.g. in Fig 4 is considered here.

Table A. Comparison between MirrorTree and GA. We report the mean and standard deviation of the TP fraction over 100 realizations of MirrorTree and of GA (see *Materials and methods*) for various datasets. GA results for 21NN and Orthology graph correspond to those of Fig 2 for HK-RR with 11.03 paralogs per species, of Fig 5A for HK-RR with 29.2 paralogs per species and of Fig C for MALG-MALK, XDHA-XDHC, LOLC-MACA and ACRE-ENVR.

	MirrorTree	GA, 21NN	GA, Orthology
HK-RR $\langle m_p \rangle = 11.03$	0.39 ± 0.02	0.60 ± 0.03	0.67 ± 0.04
HK-RR $\langle m_p \rangle = 29.2$	0.32 ± 0.02	0.34 ± 0.02	0.33 ± 0.04
MALG-MALK	0.71 ± 0.04	0.57 ± 0.05	0.81 ± 0.03
XDHA-XDHC	0.94 ± 0.01	0.76 ± 0.07	0.85 ± 0.09
LOLC-MACA	0.84 ± 0.03	0.81 ± 0.05	0.81 ± 0.04
ACRE-ENVR	0.78 ± 0.05	0.65 ± 0.09	0.61 ± 0.14

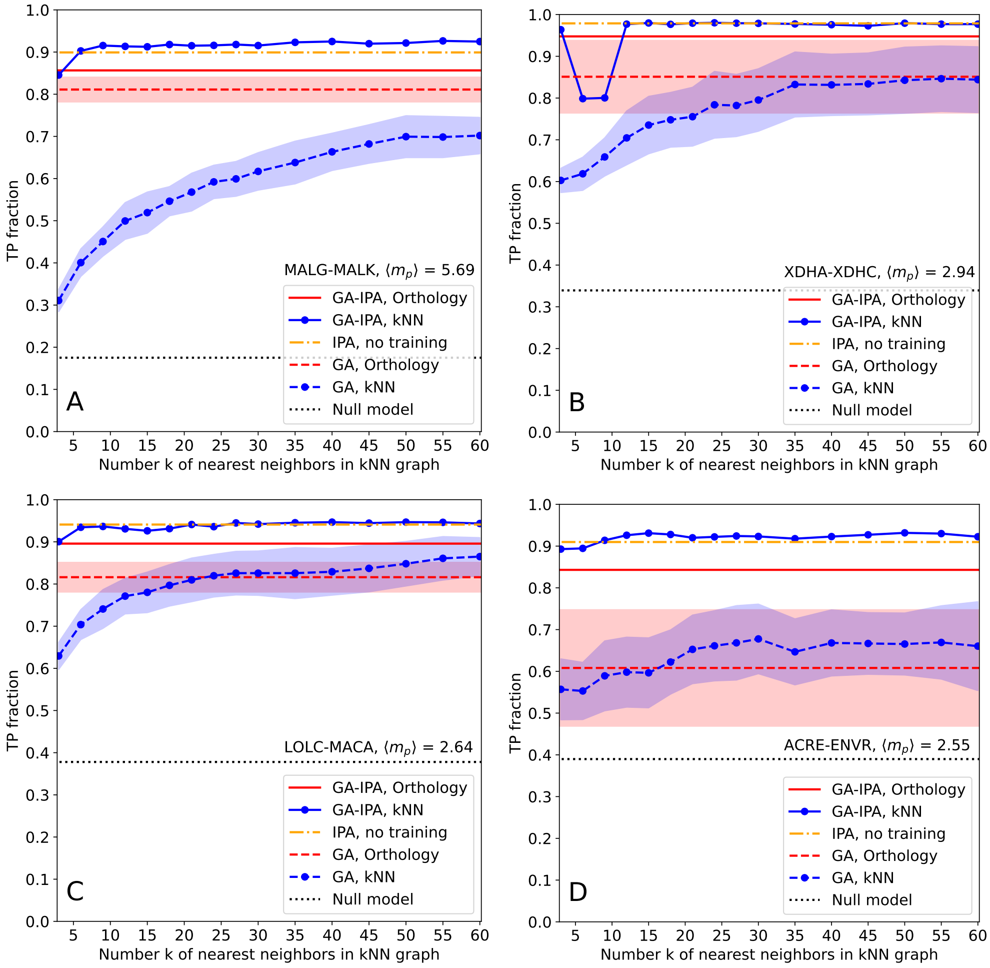

Fig C. Robust performance of GA-IPA across different data sets. The four panels (A-D) show the performances for the four datasets MALG-MALK (A), XDHA-XDHC (B), LOLC-MACA (C) and ACRE-ENVR (D), cf. *Materials and methods* for details. Recall that MALG-MALK and XDHA-XDHC are interacting pairs, while LOLC-MACA and ACRE-ENVR have no known interaction but are encoded in close proximity in prokaryotic genomes. The figures are constructed in the same way as Fig 4. In all cases, GA-IPA performs best, even if the improvement is limited in panel (A), and negligible in panels (B-D), due to the limited paralog mutiplicities in these datasets, which make IPA already very efficient without any seed co-MSA.

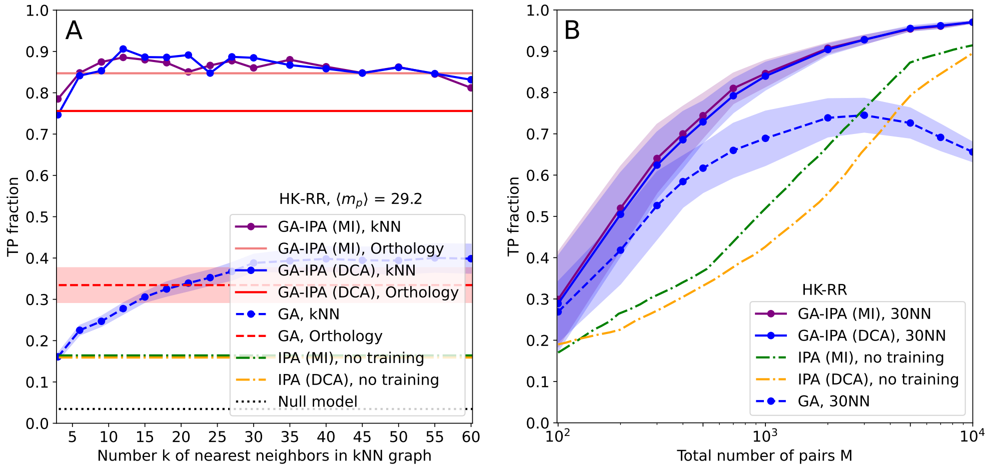

Fig D. Robust performance of GA-IPA in hard cases of paralog pairing: mutual information (MI)- versus DCA-based IPA. This figure shows the same study as Fig 5, but compares the MI-based IPA [2] to the DCA-based IPA [1] which is used in the rest of this work. (A) The mean fraction of true-positive pairings (TP ratio) is shown as a function of the number of nearest neighbors k in the kNN graph, on a dataset of HK-RR having on average 29.2 paralogs per species (same as in Fig 5A). As in Figs 4 and 5, we show results from GA-IPA (using the robust pairs obtained by GA as a seed co-MSA for IPA), and compare them to the results of GA and of IPA without seed co-MSA. For IPA (either MI- or DCA-based), we use $N_{\text{increment}} = 6$, both without (IPA) and with seed co-MSA (GA-IPA). GA-IPA achieves much larger TP fractions than GA and IPA, and similar results are obtained for MI- and DCA-based IPA. (B) Results of GA, IPA and GA-IPA for HK-RR datasets of various sizes obtained by species subsampling from the full HK-RR data set, with 11.1 paralogs per species on average (same as in Fig 5B). Both without (IPA) and with seed co-MSA (GA-IPA), the MI-based IPA slightly outperforms the DCA-based IPA, and the difference becomes smaller when using the seed co-MSA provided by GA (GA-IPA). In both cases, GA-IPA needs almost one order of magnitude less sequences than IPA to reach comparable TP fractions.

Table B. Robustness of MirrorTree and of GA. We count how many times a protein A is paired to the same protein B among 100 runs of MirrorTree and GA (see Table A and *Materials and methods*). We report the number N_{robust} of robust pairs, which are predicted across all runs, and the fraction of correctly matched pairs (TP fraction, "TP frac.") among these robust pairs.

	MirrorTree		GA, 21NN		GA, Orthology	
	N_{robust}	TP frac.	N_{robust}	TP frac.	N_{robust}	TP frac.
HK-RR $\langle m_p \rangle = 11.03$	1228	0.75	1319	0.99	875	0.97
HK-RR $\langle m_p \rangle = 29.2$	383	0.78	425	0.99	212	0.99
MALG-MALK	2108	0.87	941	0.99	3300	0.91
XDHA-XDHC	2043	0.96	919	1.00	1385	0.94
LOLC-MACA	1754	0.87	1050	0.98	1526	0.90
ACRE-ENVR	1627	0.77	638	0.97	1049	0.78

Table C. Using MirrorTree in the IPA. We consider variants of the IPA that employ MirrorTree-based scores, and compare them to our IPA results for various datasets. In the first two columns, we report the average and standard deviation of the TP fraction over 50 replicates of IPA starting from no training set, which differ in their initial random within-species pairings. We consider the IPA using DCA-based scores and a variant ("MT") using MirrorTree-based scores for pairing (see *Materials and methods*). In the rest of the table, the IPA is started from a training set. Specifically, in the next three columns, we consider the case where the DCA-based IPA starts from a training set, which is either the robust co-MSA from GA ("GA-IPA", either using GA from 21NN or Orthology graphs) or the one from MirrorTree ("MT-IPA"), see Table B. Finally, in the last column, we employ MirrorTree ("MT") both to construct the robust co-MSA and to compute scores within the IPA. We use $N_{\text{increment}} = 6$ in all cases.

	IPA (DCA), no training	IPA (MT), no training	GA-IPA, 21NN	GA-IPA, Orthology	MT-IPA (DCA)	MT-IPA (MT)
HK-RR $\langle m_p \rangle = 11.03$	0.83 ± 0.03	0.59 ± 0.03	0.90	0.86	0.70	0.55
HK-RR $\langle m_p \rangle = 29.2$	0.16 ± 0.06	0.13 ± 0.05	0.89	0.76	0.50	0.30
MALG-MALK	0.90 ± 0.01	0.82 ± 0.00	0.92	0.86	0.84	0.78
XDHA-XDHC	0.97 ± 0.00	0.95 ± 0.00	0.98	0.95	0.94	0.95
LOLC-MACA	0.94 ± 0.02	0.92 ± 0.00	0.94	0.90	0.87	0.87
ACRE-ENVR	0.91 ± 0.01	0.83 ± 0.00	0.92	0.84	0.83	0.82

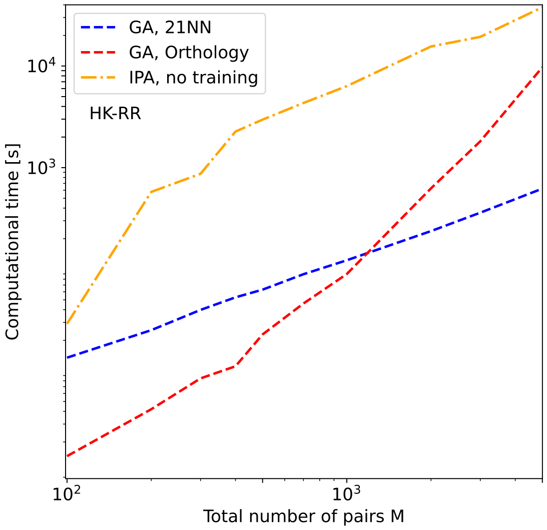

Fig E. Computational cost of GA and IPA. We show the mean computational time taken by GA and IPA versus the total dataset size (number M of sequences), for different datasets obtained by randomly subsampling species from the full HK-RR data set (as in Figs 5B and DB). These times were measured on a single core, using a laptop with an AMD Ryzen 7 4800h processor and 16 GB RAM. Note that the run time of the IPA strongly depends on M, but also on the number of iterations required, and thus on $N_{\text{increment}}$, as discussed in [1, 2]. Here we show this run time when using $N_{\text{increment}} = 6$ and starting from no training set. Due to its iterative nature, the IPA is computationally intensive. However, using a training set means that fewer iterations are required at a given value of $N_{\text{increment}}$.

References

1. Bitbol AF, Dwyer RS, Colwell LJ, Wingreen NS. Inferring interaction partners from protein sequences. Proc Natl Acad Sci USA. 2016;113(43):12180–12185.
2. Bitbol AF. Inferring interaction partners from protein sequences using mutual information. PLoS Comput Biol. 2018;14(11):e1006401.
