# Supplementary material for: Combining phylogeny and coevolution improves the inference of interaction partners among paralogous proteins
Source: PLoS Comput Biol. 2023 Mar 30;19(3):e1011010. doi: 10.1371/journal.pcbi.1011010 (PMC10089317; doi:10.1371/journal.pcbi.1011010)
Supplement: Attachment — Submitted filename: Response_reviewers_GA-IPA.pdf [file pcbi.1011010.s002.pdf]

We thank both reviewers for their overall positive evaluations of our work, and for their suggestions and comments, which have helped us to improve our manuscript. As can be seen from our point-by-point reply below, we have followed all suggestions and modified our manuscript accordingly. Note that in our reply below, the original reports are reproduced in black, while our replies are introduced in blue.

We hope that the revised manuscript meets all requirements of the reviewers, and can be published in PLoS Computational Biology.

Reviewer #1

In this work, a sequence-based method for predicting the pairings between two paralogous families of interacting proteins is presented. The method combines two previous approaches: one based on maximizing phylogeny-matching via a heuristic graph alignment procedure (GA), and another based on maximizing the inter-family residue co-evolutionary signals yielded by the paired alignments (IPA). These are combined by taking the more stable pairs generated by GA as a "seed" for IPA. The approach is tested in 4 pairs of interacting families, including the well-studied bacterial two-component system, for which the real pairing is inferred from genome co-localization. The combined approach (GA-IPA) is shown to present a higher performance compared with the original approaches for the cases with a high paralog multiplicity (many paralogs per species) and small multiple sequence alignments (low number of sequences in the families).

Predicting pairings between paralog families is important for better understanding signaling pathways and how these evolve avoiding crosstalk. Sequence-based approaches for this task are benefited from the continuous stream of new genome sequences. While it lacks conceptual novelty (just combines two existing approaches), the methodology presented here is potentially interesting as it produces better results in some particular cases. The paper is clearly written.

>>> We thank the reviewer for the encouraging assessment of our work, and for the interesting suggestions which helped us to improve our manuscript.

Major comments

My interpretation of the results is that GA-IPA only outperforms IPA for the cases with a high paralog multiplicity (many paralogs per species) and "small" multiple sequence alignments. The second is fine but the first… how realistic is that scenario? The authors "simulate" an example with 29 paralogs per specie and many species. How many of such families are out there? To demonstrate the utility of their method in the "real world" it would be desirable to quantify this (e.g. using databases of protein families/paralogs) or, at least, discuss this point in detail.

>>> Out of the almost 20,000 protein domain families in the Pfam database, more than 1300 have, on average, at least 10 paralogs per species, and about 400 more than 30 paralogs. These strongly amplified protein families contain, beyond repeat domains which are not the most relevant here, many specifically interacting protein families like receptors, transporters, kinases etc., used, e.g., in signal transduction. We are therefore convinced that this case is biologically relevant, even if specific applications go beyond the methodological scope of our manuscript. We have clarified this point in the revision, by stating the above-mentioned figures regarding Pfam families in the Introduction.

There are many other approaches for the sequence based prediction of pairings between paralogs (e.g. PMIDs 14594708, 18277381, 18215279, 22399677, 16634043, 19696150, …). The authors should include a better overview of the field in their introduction.

>>> We thank the reviewer for pointing out these highly relevant references, which we have now cited in our Introduction. In particular, it now includes the following amended sentences: "This idea has been used to discriminate interacting from non-interacting families starting from protein orthologs in the MirrorTree family of approaches [14–16], some of which address the paralog pairing problem [17 – 26]." and then "Second, interacting proteins coevolve (see [5, 27, 28] for reviews), which can be employed to predict interaction partners, as first demonstrated in a Bayesian network approach in [29]."

Related with that, it would be desirable to compare the approach presented here with some of these (unrelated) previous approaches. Comparing with the two that are combined here (GA and IPA) is fine for evaluating the added value of the combination, but for putting this new methodology in the context of the current landscape of approaches, a comparison with "unrelated" approaches would be desirable.

>>> We thank the reviewer for suggesting this comparison, which indeed puts our results in a better context. We now present a comparison of GA with a MirrorTree-based approach, and we also compare the IPA and GA-IPA results with variants based on MirrorTree, either for constructing the seed co-MSA or also in the iterative process. Overall, we find that GA performs similarly or better than MirrorTree, yields more robust seed co-MSAs, and that the coevolution-based IPA performs better than that using a MirrorTree-based pairing score. Results are shown in Tables S1, S2 and S3, and new paragraphs explaining these points have been added in Results and discussion, as well as in Materials and methods. Note that most of these papers were published without code, so we had to reimplement part of the methods ourselves.

Figure 1 is a little bit misleading. In real families orthologs are more self-similar than paralogs. The clades in the trees should correspond to orthologous groups (i.e. mixed colors/species).

>>> This figure is intended to illustrate the main principles of the method, and not to represent the real relations present in any gene family. While we agree that in most cases orthologs from related species are closer than paralogs in the same species, highly amplified protein families contain examples of recent duplications. To avoid confusions, we clarify this point in the caption of Fig. 1, by stating: "We show schematics to illustrate the key points of our approach (these schematics are conceptual and do not represent actual phylogenetic trees or similarity networks)."

We also now make more explicit in *Materials and methods* that kNN networks may contain intra-species links between paralogs. Specifically, we have amended the following sentences: "kNN network: In this network, each node is connected to its k nearest neighbors, possibly including links inside one species (connecting closely related paralogs), or multiple links between species." and: "Contrary to kNN networks, orthology networks do not contain any links inside species, even when close paralogy is present."

Minor

Requiring two families with the same number of members in each species is really a drawback and, related to the comment above on the paralog multiplicity, I do not know how many cases of these are in the real world (specially in eukarya). The authors acknowledge this and say that their method could be modified to work with different number of members. While doing that is beyond the scope of this particular work, at least giving some receipts on how to apply the current method in those cases should be given: i.e. what can I do if I want to apply this method to my families with different #proteins? Which ones could I discard?....

>>> We agree with the reviewer that assuming the same number of members in each species is a limitation. We have made our discussion of this point more explicit, including specific points on how to deal with the case where there are different numbers of members. The Conclusion now reads:

“Here, for simplicity, we considered the simple case where each species comprises the same number of paralogs from each of the two families. However, in natural data, species include different number of paralogs from different families. In [33], this was addressed by using an injective pairing from the family with fewer paralogs into the family with more paralogs. Specifically, during the iterative procedure, in a species with more proteins A than proteins B, each protein B is matched to one protein A so that the pairing score is maximized, and one discards the remaining proteins A. This only requires a minor change in our algorithm and could be employed in GA-IPA.”

It is not true that most co-evolutionary studies “disregard phylogeny” (Conclusions). Indeed, the new-wave of method for detecting residue co-evolution are characterized, among other things, for correcting the phylogenetic signal in one way or another (in most cases not explicitly but implicitly).

>>> We apologize for this misleading wording. We have clarified this sentence, which now reads: “Conversely, most coevolutionary studies effectively treat sequences as close to independent, and employ corrections to reduce the impact of phylogenetic correlations that could obscure functional coevolution.”

Reviewer #2

The paper by Gandarilla-Perez et al. presents an interesting and novel computational framework to predict protein-protein interactions from Multiple Sequence Alignments (MSA). The methods proposed aims at identifying pairs of interacting proteins exploiting the inter-protein evolutionary correlations observed among their residues. While in previous approaches, presented by some of the authors, such evolutionary correlations were quantified using residue-residue correlations only (by means of Mutual Information (MI) and Direct Coupling Analysis (DCA)), here the aim is to integrate this information with phylogenetic correlations, which were not expected to be captured by MI and DCA.
Phylogenetic correlations are then used to produce partial co-alignments of interacting proteins (co-MSAs), obtained from similarity graph alignment, for which authors developed a stochastic algorithm based on simulated annealing to solve the graph-alignment (GA) problem. The co-MSA is the used as an input to the iterative pairing algorithm (IPA) based on residue coevolution as detected by DCA (or by MI).
I find the algorithmic approach presented in the paper to be technically sound and well grounded on protein evolution concepts.

>>> We thank the reviewer for the positive assessment of our work and for the interesting suggestion.

As for the results presented, the combination of GA+IPA clearly display improved performances of interaction partner inference when the average number of potential partners is large and when the total data set size is modest (see Fig5 and related results). However, in (arguably) more "standard" situations the gain of this new approach vs already existing (and published) approaches is very marginal (Fig.4-Fig.S4 and related results). For this reason, I think authors should address the following major point:

- what is the computational cost of the GA algorithm? How does it scale w.r.t. key parameters (such as the total number of sequences or the average multiplicities)?. Here I'd be only interested with a computational analysis (say execution time vs N or similar).

In other words, I think it would be important for the new approach to be actually deployed in a systematic way, to know whether the computational cost is worthy the (possible) little gain in the accuracy of the inference.

>>> We agree with the reviewer's interpretation of our results, and we have added a new figure (Fig S5) to show how computational time depends on dataset size for GA and for IPA. An associated paragraph has also been added at the end of *Results and discussion*: "Computational time. GA is usually less computationally costly than IPA (see Fig. S5). Furthermore, using a training set reduces the number of required IPA iterations compared to starting from no training set. However, in order to get a robust seed co-MSA, we employ multiple runs of GA, which increases the computational cost of the GA step, but is amenable to naive parallelism. Overall, our results show that using GA to provide a training set for the IPA yields important performance gains in difficult cases where there are few sequences or many paralogs per species, while IPA is already very good in other cases. Therefore, with computational cost in mind, we recommend the use of GA primarily in such difficult cases."

Note that, in response to the first reviewer, we have also included some statistics about the paralog numbers in highly amplified protein families: Out of the almost 20,000 protein domain families in the Pfam database, more than 1300 have, on average, at least 10 paralogs per species, and about 400 more than 30 paralogs. These strongly amplified protein families contain, beyond repeat domains which are not the most relevant here, many specifically interacting protein families like receptors, transporters, kinases etc., used, e.g., in signal transduction. We are therefore convinced that this case is biologically relevant, even if specific applications go beyond the methodological scope of our manuscript. We have clarified this point in the revision.
