# Supplementary material for: Combining phylogeny and coevolution improves the inference of interaction partners among paralogous proteins
Source: PLoS Comput Biol. 2023 Mar 30;19(3):e1011010. doi: 10.1371/journal.pcbi.1011010 (PMC10089317; doi:10.1371/journal.pcbi.1011010)
Supplement: Attachment — Submitted filename: Response_reviewers_GA-IPA.pdf [file pcbi.1011010.s003.pdf]

We thank both reviewers for their positive evaluations of our revisions. We have changed the last point raised by Reviewer #1, cf. below. Note that in our reply below, the original reports are reproduced in black, while our replies are introduced in blue.

We hope that the revised manuscript meets all requirements of the reviewers, and can be published in PLoS Computational Biology.

Reviewer #1: The authors addressed all may concerns and performed new experiments. I think the paper is clearer now and can be published.

My only (minor) concern is that, as stated now in the "Comparison with MirrorTree-based methods" it looks like these mirrirtree-based approaches for paralog mapping use Hamming distances for quantifying protein similarities. This is not true and they use other measures (average similarity, substitutions/site, ...) I understand the authors' reason to implement mirrortree approaches with Hamming distances in the context of this work as those are the ones used by their method, and, as commented in other part of the manuscript, I would not expect major differences using other measures (so I do not think an implementation of the approaches with their "original" distances is required). But at least the point should be clarified and an explanation why mt approaches were implemented with hamming distances for this particular work added.

We are happy that our modifications meet all concerns of the reviewer. We also totally agree that our description of the distance measures used in mirror-tree based approaches were too simplified, we have elaborated this point in the *Methods* section of our paper.

Reviewer #2: The authors have addressed all my remarks in a thorough and clear manner. Overall, the manuscript is very well written and contains interesting results which are well supported by the presented evidence.
I recommend the manuscript for publication.

We are happy that our modifications meet all concerns of the reviewer.
